# Identification and Validation of Reference Genes for RT-qPCR Analysis in Switchgrass under Heavy Metal Stresses

**DOI:** 10.3390/genes11050502

**Published:** 2020-05-03

**Authors:** Junming Zhao, Man Zhou, Yu Meng

**Affiliations:** 1 Department of Grassland Science, Sichuan Agricultural University, Chengdu 611130, China; junmingzhao163@163.com; 2 Zonation Fringe Technology Co., Metro Vancouver, BC V5C 2A0, Canada; zonation.fringe.technology.co@gmail.com; 3 College of Science and Technology, Wenzhou-Kean University, Wenzhou 325060, China

**Keywords:** reference genes, *Panicum Virgatum* L., heavy metal stresses, real-time quantitative PCR

## Abstract

Switchgrass (*Panicum Virgatum* L.) has been recognized as the new energy plant, which makes it ideal for the development of phytoremediation on heavy metal contamination in soils with great potential. This study aimed to screen the best internal reference genes for the real-time quantitative PCR (RT-qPCR) in leaves and roots of switchgrass for investigating its response to various heavy metals, such as cadmium (Cd), lead (Pb), mercury (Hg), chromium (Cr), and arsenic (As). The stability of fourteen candidate reference genes was evaluated by BestKeeper, GeNorm, NormFinder, and RefFinder software. Our results identified *U2AF* as the best reference gene in Cd, Hg, Cr, and As treated leaves as well as in Hg, Pb, As, and Cr stressed root tissues. In Pb treated leaf tissues, *18S rRNA* was demonstrated to be the best reference gene. *CYP5* was determined to be the optimal reference gene in Cd treated root tissues. The least stable reference gene was identified to be *CYP2* in all tested samples except for root tissues stressed by Pb. To further validate the initial screening results, we used the different sets of combinatory internal reference genes to analyze the expression of two metal transport associated genes (*PvZIP4* and *PvPDB8*) in young leaves and roots of switchgrass. Our results demonstrated that the relative expression of the target genes consistently changed during the treatment when *CYP5/UBQ1*, *U2AF/ACT12*, *eEF1a/U2AF,* or *18S rRNA/ACT12* were combined as the internal reference genes. However, the time-dependent change pattern of the target genes was significantly altered when *CYP2* was used as the internal reference gene. Therefore, the selection of the internal reference genes appropriate for specific experimental conditions is critical to ensure the accuracy and reliability of RT-qPCR. Our findings established a solid foundation to further study the gene regulatory network of switchgrass in response to heavy metal stress.

## 1. Introduction

Real-time quantitative PCR (RT-qPCR) has become the leading technique applied in gene expression analysis due to its advantageous characteristics, such as high-throughput, high sensitivity, and specificity, along with great repeatability. Reference genes with stable expression levels are used as the standard markers to calibrate and ensure the accuracy and validity of results from RT-qPCR [1,2]. Thus, a few conventional housekeeping genes, such as *β-Actin* and *18S rRNA,* are commonly used as reference genes in RT-qPCR for plants [3]. However, based on up-to-date studies, the universal reference gene with stable expression profiles in different tissues and organs, developmental stages as well as experimental conditions has not been discovered. Therefore, it is of pivotal importance to identify the appropriate and stable reference genes associated with various situations in the analysis of gene expression profiles.

Heavy metal, as the main environmental pollutant, has raised a growing concern in ecological and global public health in recent years [4]. With the overdevelopment and excessive utilization of mineral resources and reserves, the disposal of electronic waste, and the extensive application of pesticides and fertilizers, the pollution in soil and water by heavy metals has become an increasingly serious problem [5]. Furthermore, the enrichment of heavy metals in the soil by agronomic crops endangers human health in a direct manner. Therefore, the remediation of heavy metal polluted soils, especially phytoremediation, has gained increasing attention from both academia and industries due to its lower cost and fewer side effects than conventional chemical and physical techniques [6]. Though the hyperaccumulator has become the hotspot for ecological restoration in recent years, utilization of energy plants for remedying heavy metal contaminated soil can achieve a win–win situation for the production of biomass raw materials as well as the management of the polluted soil [7].

Switchgrass (*Panicum Virgatum* L.), a C4 warm-season perennial grass species, is originated from North America and widely distributed in non-natural areas (above 55° north latitude) [8]. The C4 grass has many merits, such as high efficiency in photosynthesis, high utilization of nitrogen, water, and nutrients, and it is also effective in water–soil conservation as well as the increase in organic matter in the soil [9]. Generally, the full growth season of switchgrass starts in the third year after plantation, and a single plantation lasts from 10 to 15 years. In addition, switchgrass is well tolerated and grows well on the land under a variety of stress conditions, such as drought, alkali-salt, flooding, and leanness [10]. The renewable energy that is produced from switchgrass has been reported to be about five fold of the energy that is consumed during the production process [11]. In addition, considering the environmental benefits in soil conservation and reducing greenhouse gas emissions, switchgrass has been recognized as the new energy plant with great potential, which makes it ideal for the development of phytoremediation on heavy metal contamination in soils [7,10].

In this study, we have selected fourteen common housekeeping genes as candidate reference genes from previous studies [10,12], aiming to identify appropriate reference genes with stable expression in various tissues of switchgrass and analyze its response to stress induced by different heavy metals. We further studied the differential gene expression of *PvZIP4* and *PvPDR8* from Zinc/Iron regulatory transporter family (ZIP) and ATP-binding cassette transporter family (ABC), respectively, pre and post the stress. Our results not only facilitate the understanding of the molecular mechanism of the heavy metal stress-induced response in switchgrass, but also establish the foundation for further studies on remediation of heavy metal contamination in soil.

## 2. Materials and Methods

### 2.1. Plant Materials and Treatment

The switchgrass cultivars “Alamo” seeds were sterilized in 10% NaClO for 30 min. After five times rinsing with deionized water, 100 seeds were sown and germinated in trays (30 cm length, 16 cm width, and 12 cm deep) with 1/2 strength Hoagland’s solution. Plants were incubated in a growth room with the following environmental conditions: temperature 25 °C/20 °C (12 h day/12 h night), photosynthetically active radiation 400 μmol m^−2^s^−1^. Two months after plantation, plants were treated with 1 mM solution of cadmium (Cd), lead (Pb), mercury (Hg), chromium (Cr), and arsenic (As), respectively. Three replicates (three pots) were conducted for each heavy metal treatment group in a completely randomized design. Leaf and/or root samples were collected at the following time points 0, 1.5, 3, 6, 12, 24, and 48 h post-treatment with different heavy metals. Tissue samples were stored at −80 °C for further analysis.

### 2.2. Extraction of Total RNA and Reverse Transcription

Switchgrass tissues weighing 50–100 mg were pulverized using a Tissuelyzer (Qiagen, Germantown, MD, USA), and total RNA was extracted via Trizol reagent (Invitrogen, Carlsbad, CA, USA). The quality of RNA was validated by running the 1.2% agarose gel electrophoresis and visualized by an Analytikjena ScanDrop (Jena, Germany). Total RNA (1 µg) was extracted for each sample and reverse transcribed into the first strand of cDNA using the PrimeScript^TM^ RT reagent kit (TaKaRa, Dalian, China) and stored at −80 °C for further analysis.

### 2.3. Design and Validation of Specific Primers

Fourteen candidate reference genes were selected based on previous studies [10,12]. These genes are as follows: *18Sr RNA*(GR878775), *ACT12*(GR878265), *ACT2*(FL724919), *CYP2*(FL942644), *CYP5*(FE633090), *eEF1a*(GR876801), *eEF4a*(GR877213), *U2AF*(FL907910), *UBC*(GR879761), *FTSH4*(FL791612), *UBQ1*(FL955474), *UBQ2*(FL920273.1), *UBQ6*(FE609298), and *UCE*(GR879053). *PvZIP4*(Pavir.J08901) from the ZIP family and *PvPDR8*(PTHR19241) from the ABC family in switchgrass were utilized to validate selected reference genes in further gene expression analysis. Specific primers were designed via the Primer Premier 5.0 software and synthesized by Qingke Biotech (Chengdu, China). The sequences of primers for candidate reference genes and transporter genes in RT-qPCR are listed in Table S1. The specificity of primers was validated from the melting curve of RT-qPCR reaction.

### 2.4. Real-Time PCR

Quantitative analysis via real-time PCR was conducted using a Sosofast Supermix reagent kit (Bio-Rad, Hercules, CA, USA) and the CFX96 real-time PCR system (Bio-Rad, Hercules, CA, USA). The experiment was performed in 20 μL volume of reaction in ice-bath. Reaction reagents were as follows: 1 μL of primer at a final concentration of 0.2 μmol·L^−1^, 10 μL qPCR SYBR Green SuperMix, 2 μL cDNA, and ddH_2_O to bring the total volume to 20 μL. The sequential steps of real-time PCR include pre-denaturation at 94 °C for 10 s, denaturation at 94 °C for 10 s, annealing at 62 °C for 5 s, 40 cycles in total. Three technical replicates were performed in a sample mixture with each heavy metal stress at each time point. Then the two closest cycle threshold (Ct) values were used for RT-qPCR analysis.

### 2.5. Analysis of Stability

The cycle threshold (Ct) value for each reference gene was obtained from RT-qPCR and analyzed through GeNorm [13], NormFinder [14], BestKeeper [15], and RefFinder (http://www.leonxie.com/referencegene.php) software. When the data analysis was performed with GeNorm and NormFinder, the Ct value was first converted to relative quantitative Q value via the formula Q = 2^−ΔCt^ (ΔCt = Ct_sample_ − Ct_min_). Ct_sample_ is the Ct value of the housekeeping gene in each of heavy metals treatment; Ct _min_ indicates the lowest Ct value of this housekeeping gene among each of heavy metals treatment. Then the expression stability measurement (M) value was calculated by the GeNorm program for each candidate reference gene. BestKeeper directly utilized the Ct value for stability analysis without the additional converting step to measure the comparisons of the coefficient of variance (CV) and the standard deviation (SD). Finally, RefFinder integrates all three methods mentioned above to calculate the geometric mean for each reference gene and the comprehensive ranking index of stability. A lower index value indicates a higher stability of the reference gene. The optimal number of reference gene was determined by the paired coefficient of variation V_n_/V_n+1_. It is generally considered that when the value of V_n_/V_n+1_ is less than 0.15, it is unnecessary to introduce a new reference gene. Otherwise, the (n+1)^th^ reference gene is in need.

### 2.6. Validation of Reference Genes by Expression Analysis of Two Metal Transporters Genes

The two homologs of metal transporters *PvZIP4* and *PvPDR8* from switchgrass were obtained from the database (https://phytozome.jgi.doe.gov/pz/portal.html). For the validation of selected reference genes, the expression levels of two genes were analyzed using the most and least stable reference genes under heavy metal stress, calculated using the 2^−ΔΔCt^ method [16]. Three replicate samples were included for each treatment, and three technical replicates were conducted for each biological sample.

## 3. Results

### 3.1. Specificity of Primers for Reference Genes

The RT-qPCR reactions were performed using the total RNA reverse transcription products from young leaves and young roots of switchgrass treated by different heavy metals treatment as the template. The results suggested that a distinct single peak was identified in the melting curves of all genes (Figure S1). In addition, the PCR amplification curve of all samples showed great repeatability, indicating that the primers were able to amplify the desired products of each gene with high specificity and no primer dimer. Therefore, our results from RT-qPCR were confirmed to be valid and reliable.

### 3.2. Analysis of Reference Gene Expression

**Figure 1:**
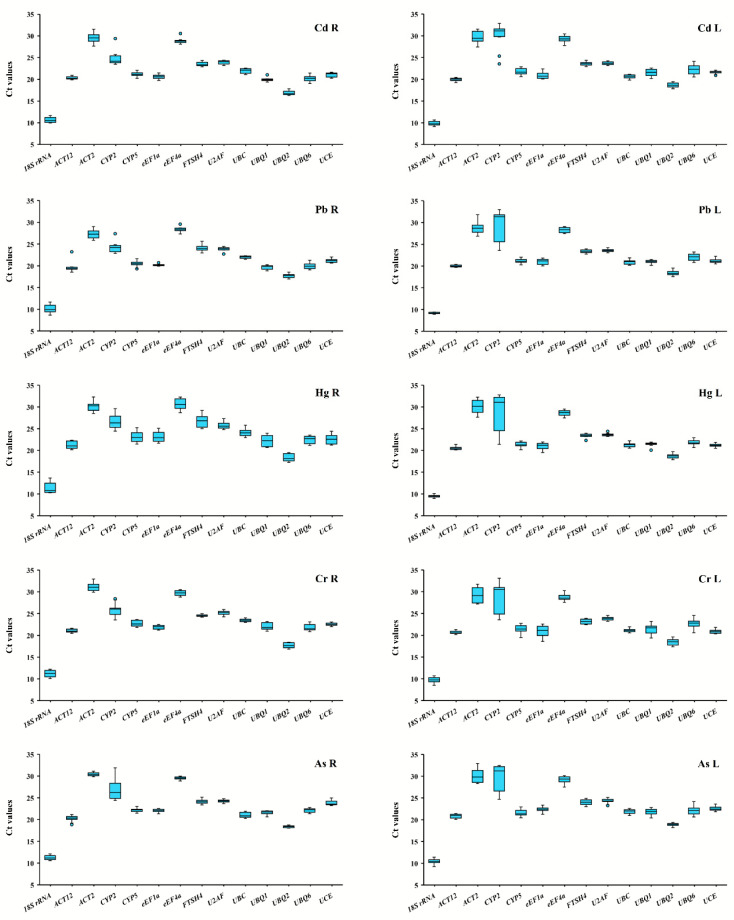
Median cycle threshold (Ct) values for fourteen candidate reference genes in switchgrass root and leaf samples under heavy metals stress conditions. The variation is displayed as medians values (lines across the box plot), 25th to 75th percentiles (boxes), and the maximum and minimum values (whiskers). Cadmium-treated leaves (CdL) and roots (CdR); lead-treated leaves (PbL) and roots (PbR); mercury-treated leaves (HgL) and roots (HgR); chromium-treated leaves (CrL) and roots (CrR); arsenic-treated leaves (AsL) and roots (AsR), the same below.

It has been reported that the Ct value of reference genes is inversely proportional to the expression level of that gene [17]. The greater the Ct value of the reference gene is, the lower the amount of the target gene being expressed in the sample and vice versa. Expression abundance of fourteen reference genes in all samples was analyzed via RT-qPCR (Figure 1; Table S2). Our results demonstrated that the Ct value for each reference gene was in the range of 5–33. The lowest Ct value was found in the *18S rRNA* gene ranging between 5 and 15, while its expression abundance was the highest among all the reference genes. The low values were in the case of *UBQ2* and *ACT12*. *ACT2* had high value as *CYP2* and *eEF4a*, indicating the lowest expression abundance. The large distribution of Ct values suggested that the expression abundance differs among the reference genes.

### 3.3. Analysis of Reference Genes Stability

#### 3.3.1. GeNorm Analysis

**Figure 2:**
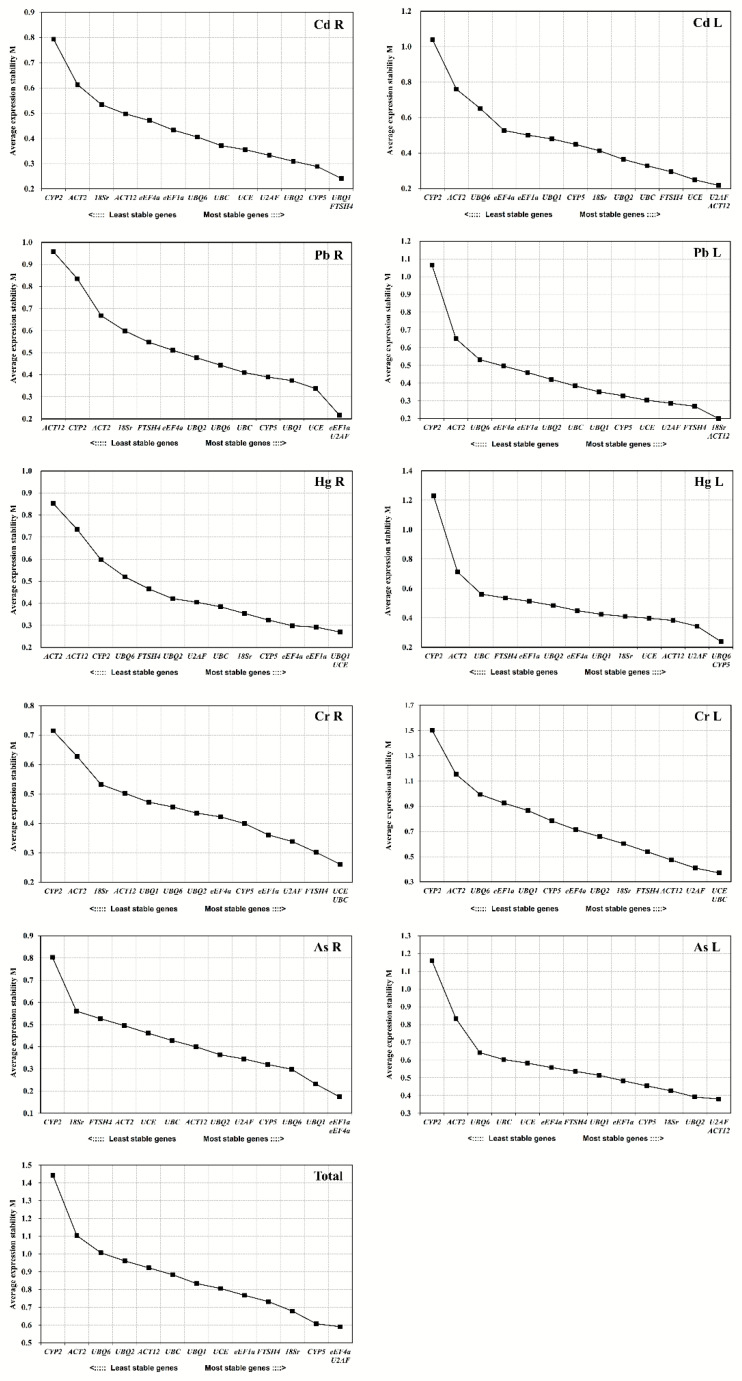
Expression stability measurement (M) for fourteen candidate reference genes in switchgrass root and leaf samples under heavy metals stress conditions.

The expression stability of reference genes was analyzed via GeNorm V3.4 and represented by calculated M values. The lower the M value is, the higher stability the reference gene has and vice versa. Based on this principle, the M value was determined for each reference gene of all samples. Different combination of reference genes was shown to be the most stable ones in roots and leaves responding to each heavy metal stress: *UBQ1/FTSH4* in Cd-treated roots (CdR) *U2AF/ACT12* in Cd-treated leaves (CdL), *eEF1a/U2AF* in Pb-treated roots (PbR), *18Sr/ACT12* in Pb-treated leaves (PbL), *UBQ1/UCE* in Hg-treated roots (HgR), *UBQ6/CYP5* in Hg-treated leaves (HgL), *UCE/UBC* in Cr-treated roots (CrR), *UCE/UBC* in Cr-treated leaves (CrL), *eEF1a/eEF4a* in As-treated roots (AsR) and *U2AF/ACT12* in As-treated leaves (AsL). However, the overall evaluation suggested that *eEF4a* and *U2AF* in both leaf and root tissues displayed the highest stability with the lowest M values under all stress conditions tested (Figure 2).

**Figure 3:**
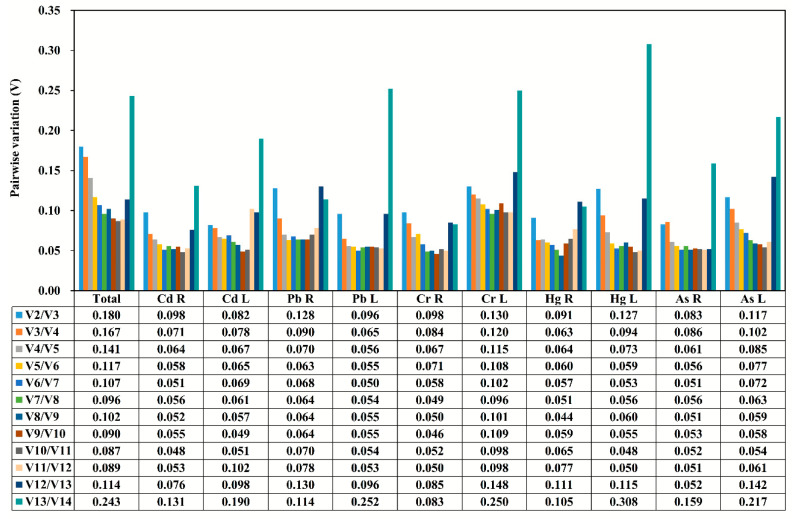
The pairwise variation (V) measure of the candidate reference genes using GeNorm. V_n_/V_n+1_ values were used to calculate the optimal number of reference genes (*n*).

When the pairwise variation V_n_/V_n+1_ value is lower than the threshold of 0.15, the value (n) can be considered as the optimal number of reference genes for accurate normalization [13]. The V_2/3_ value of reference gene in all samples under the stress of each heavy metals was shown to be smaller than the threshold value 0.15 (Figure 3), indicating that the gene expression analysis needs two reference genes to achieve the best performance. However, the combined use of the four reference genes could be suitable for testing all the considered tissues and stress conditions.

#### 3.3.2. BestKeeper Analysis

The standard deviation (SD) of the Ct value of each housekeeping gene was calculated via BestKeeper V1. With the SD value less than 1, the housekeeping gene is considered as the stable one. Furthermore, the lower the SD value is, the higher stability that gene displays. Reversely, the gene was counted as being not stable if the SD value is higher than 1. Our results demonstrated that the *U2AF* gene exhibited the highest stability in total, particularly in the root sample treated with Hg and leaf samples treated with Pb, Hg, and As (Table 1). *UCE* was found to be the most stable reference gene in leaf samples treated with Cd, while *eEF4a* was identified to be the most stable reference gene for root samples treated with Cd and As. In the root and leaf samples treated with Pb and Cr, respectively, the *UBC* gene was shown to have the highest stability. When the root samples were treated with Cr, *FTSH4* was found to be the most stable gene. Among all of the reference genes, the SD values of the *CYP2* gene were demonstrated to be greater than 1 in all treatments except for PbR, indicating the instability of this gene (Table 1).

#### 3.3.3. NormFinder Analysis

The lower value calculated from NormFinder V20 indicates the higher stability of the housekeeping gene expression. Results demonstrated that *U2AF* was shown to be the most stable reference gene in leaf samples under stress by Cd and root samples under stress by Hg, Cr, and As (Table 2). *CYP5* was ranked as the most stable reference gene in root tissues treated with Cd. In response to Pb stress, *18S rRNA* and *eEF1a* were identified to have the highest stability in both leaf and root tissues. In the leaf samples treated with Hg and Cr, the *ACT12* gene was identified to have the highest stability. *UBQ2* was shown to be the most stable reference gene in leaves treated with As. Overall evaluation identified four reference genes to be the most stable ones as follows: *U2AF* (0.463), *CYP5* (0.484), *UBQ1* (0.539), *eEF4a* (0.569). *CYP2* was shown to be the least stable gene in all samples except for the root tissues treated with Pb and Hg (Table 2).

#### 3.3.4. RefFinder Analysis

RefFinder V1.0 was used to evaluate the comprehensive stability of reference genes integrating the methodologies of GeNorm, NormFinder, and BestKeeper analyses. Our results identified *U2AF* along with different genes to be the ideal reference genes in leaf tissues treated with Cd, Hg, Cr, As, and as well as root tissues in response to Pb, Hg, Cr, and As stress (Table 3). *18S rRNA* and *ACT12* were found to be optimal reference genes in Pb treated leaf tissues. In Cd treated root samples, *CYP5* and *UBQ1* were demonstrated to be the appropriate reference genes. Regarding the unstable reference genes, *CYP2* was shown to have the least stability in all treatments except for root tissues with Pb. Furthermore, *ACT2* was found to have poor stability in all samples except for root tissues treated with Pb and As (Table 3).

### 3.4. Detection of Target Gene Expression Levels Normalized by Screened Reference Genes

**Figure 4:**
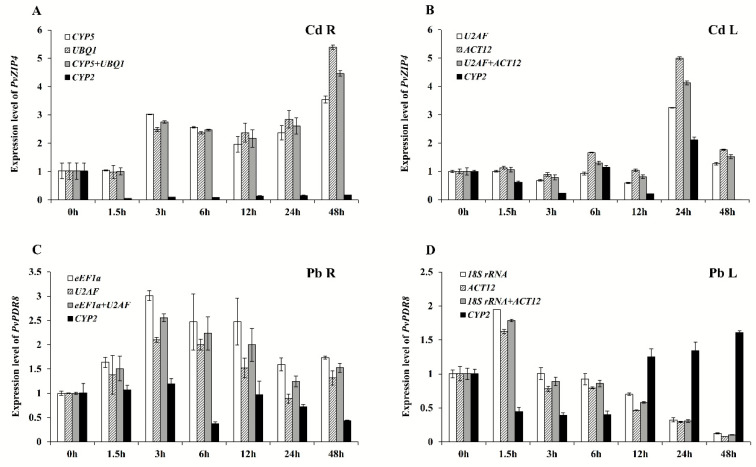
Expression levels of *PvZIP4* and *PvPDR8* in switchgrass leaves and roots under Cd and Pb stresses at different times. (**A**,**B**) represent expression levels of *PvZIP4*. (**C**,**D**) represent expression levels of *PvPDR8*. The relative expression levels were fold-change to time 0 h. R, L represent roots and leaves, respectively. Bars indicate standard errors.

Recent studies have identified numerous genes involved in heavy metal stress response. The expressions of these genes were shown to function significantly in cellular transportation and enrichment of heavy metals as well as enhancement of plant resistance [18,19]. Especially gene coded proteins involved in the transportation of heavy metals have been extensively studied. These genes include the ABC transporter family, ZIP family, and heavy-metal ATPases (HMA) family, and they are reported to improve the tolerance towards heavy metals in plants [20,21,22]. Based on the comprehensive evaluation of reference genes stability in young leaves and young roots of switchgrass in response to different heavy metals, we selected and combined the reference genes with the least and highest stability to analyze the expression of *PvZIP4* gene from ZIP family and *PvPDR8* gene from ABC family in leaf and root tissues to validate our candidate reference genes (Figure 4). When *CYP5/UBQ1* or *U2AF/ACT12* was used as the reference gene pair, the change in relative expressions of target genes in response to Cd treatment was basically consistent. Meanwhile, the expression profile of target genes under Pb stress was also consistent when normalized by *eEF1a/U2AF* or *18S rRNA/ACT12*, indicating that the expression of these reference genes is stable (Figure 4). However, when *CYP2* was used as the reference gene, a significant decrease in the relative expression of *PvZIP4* in Cd treated root samples shown at 3 h, 6 h, 12 h, 24 h, and 48 h compared to 0 h, was observed, which contradicted with the increasing trend demonstrated by other reference genes. Moreover, by using the *CYP2* as the reference gene, the expression of *PvPDR8* increased in leaves after 12, 24, and 48 h of Pb treatment compared to 1.5h, 3h, and 6h, as opposed to the time-dependent expression change pattern obtained from the reference genes *18S rRNA, ACT12*, or the pair (*18S rRNA/ACT12)*. Our results suggested that *CYP2* had the poor stability of expression in young leaf tissues with different treatments, making it not appropriate to adjust the relative expression of target genes in an accurate manner.

## 4. Discussion

The selection of proper internal reference genes largely depends on the target tissues, cells, and experimental conditions. The internal reference genes display specific differential expression when tissues/organs, developmental stages, and biotic/abiotic stress differ. There is no single internal reference gene which is constantly stable in expression under different experimental conditions in plants. The stability of the conserved reference genes also differs in different plant species. Take the internal reference gene *GAPDH* as an example, it displays poor expression stability in crops, such as wheat (*Triticum aestivum* L.) [23] and chicory (*Cichorium intybus* L.) [24], while being stable in grape (*Vitis vinifera* L.) [25] and sugarcane (*Saccharum officinarum* L.) [26]. Nakayama et al. [27] identified that *Fbox/60s* and *Fbox/ABC* are proper internal reference genes in the seedling tissue of soybean (*Glycine max*), while *ELF1B* and *ACTB* are identified as appropriate internal reference genes in soybean root tissues. It supports the argument that the internal reference genes display differential expression in different species and tissues. The previous studies showed that the expression of internal reference genes is closely related to experimental conditions. *TIP41* in *Arabidopsis thaliana* L. was stable in expression under the nutrient deficient stress [28], while its expression stability was significantly reduced when the stress was induced by copper and cadmium [29]. In addition, the stability of the reference genes from the same gene family differs. Gutierrez et al. [30] discovered that the expression of the internal reference gene *UBQ5* was more stable in *Arabidopsis* than *UBQ4*, *UBQ10*, and *UBQ11* though they are from the same gene family. Therefore, it is imperative to select the stable reference gene based on the specific experimental conditions for RT-qPCR.

Switchgrass has gained increasing popularity in the study of energy plants in recent years. The previous studies focused on the expression analysis of internal reference genes in switchgrass under various abiotic stress conditions, such as drought, salinity, high temperature, and water flooding [10,12]. However, it did not delve into the systemic comparative analysis of expression stability of these internal reference genes among different tissues of switchgrass under heavy metal stress, resulting in the possible deviations in quantifications of the expression of target genes in response to different heavy metal treatments. Housekeeping genes are often expressed in cells with an active metabolism, which maintain the basic functions of cells and play an important role in the regulation of the cell cycle. Housekeeping genes are better candidate genes for evaluating gene expression and its functional properties [31]. Commonly used housekeeping genes in Gramineae crop, e.g., actin (*ACT*), 18S ribosomal RNA protein (*18S rRNA*), cyclophilin (*CYP*), eukariotic elongation factor (*eEF*), splicing factor U2af (*U2AF*), ftsh protease (*FTSH*), ubiqutin (*UBQ*), and ubiqutin conjugating enzyme (*UCE*) are considered to be suitable due to their presence in all nucleated cell types and essential functions in cell survival. Moreover, their expression has been considered to be stable in various tissues [32,33]. Our study reports the first validation of housekeeping genes in switchgrass allowing the identification of the most suitable reference gene(s) for normalization of RT-qPCR in different plant tissues (roots and leaves) and different time-courses subjected to heavy metal treatments such as by Cd, Pb, Hg, Cr, and As.

To avoid the limitations of using only single software analysis, our study applied three analytical approaches GeNorm, NormFinder, and BestKeeper to determine the expression stability of internal reference genes in different tissues under heavy metal stresses. The basis for evaluating gene stability in GeNorm is to use the logarithmic conversion value (2^−ΔCt^) of each gene to calculate the average variability (M value) [13]. Meanwhile, GeNorm can determine the optimal number of reference genes required for quantitative analyses: When the comparative difference analysis is performed on the internal reference gene normalization factor (V_n_/V_n+1_), the n value equals the number of optimal reference genes applied in RT-qPCR analysis. In this study, the gene expression analysis needs two reference genes to achieve the best performance. The combined use of the four house-keeping genes could be suitable for testing all the considered tissues, and stress conditions were based on the pairwise variation V_n_/V_n+1_ value. However, considering we commonly select only single heavy metal treatment to study the gene regulatory network of plants in response to heavy metal stress, four reference genes for gene normalization in all the considered tissues and stress conditions have limited practical values. The algorithm of NormFinder is similar to GeNorm using the logarithmic conversion value (2^−ΔCt^) as the relative expression of the gene to calculate the stability of gene expression [14]. BestKeeper focuses on the standard coefficient variation (SD) and variation correlation coefficient (CV) to screen the stability of internal reference genes [15]. The results from these approaches differ due to the algorithm differences. For example, *U2AF* was determined to be the most stable reference gene by GeNorm and NormFinder. However, it turned out that *ACT12* was evaluated as the best in BestKeeper analysis. In addition, GeNorm alone suggested that *eEF4a* and *U2AF* were the top selections with the highest stability. The result inconsistency has been reported previously when different analytical software was applied [34]. Therefore, RefFinder integrates the algorithms of GeNorm, NormFinder, and BestKeeper to achieve comprehensive evaluation of the stability of reference genes, avoiding the unilateral judgment from a single method. In this study, *U2AF* displayed the highest stability in Cd, Hg, Cr, and As treated leaves as well as in Pb, Hg, Cr, and As treated roots of switchgrass. *U2AF* has been reported in other studies to be the most stable reference gene, such as in *Pinus massoniana* L., at different stages post-inoculation by nematode [35] and roots/leaves of *Paspalum vaginatum* Sw. under Cd and cold stress [36]. In the roots of switchgrass subjected to Cd treatment, *CYP5* and *UBQ1* were determined to be the most appropriate internal reference genes. *CYP5* was identified as the most stable reference gene in ganoderma under various experimental conditions [37]. De Andrade et al. [38] found that *UBQ1* was stably expressed in leaves of *Saccharum* spp under the drought stress. *18S rRNA* displayed the best stability in Pb treated leaves of switchgrass.

Early selection of internal reference genes is mainly dependent on the assumptions of the essentiality of the housekeeping genes’ functions. For example, based on the essential functions of *Actin* and *TUB* in cytoskeleton composition, these genes were speculated to be stably expressed in all cellular and physiological states [39]. However, the stability of internal reference genes can actually vary in different conditions in reality [40]. *Actin2* in our study had poor stability under all experimental conditions. Some previous studies claimed that *Actin* was not the proper internal reference gene in chrysanthemum under abiotic stresses (high temperature, water flooding, aphid) [41] and wild type potato before and after the cold acclimation [42]. However, *ACT12*, another member of the *Actin* gene family, was shown to be stably expressed in leaves of switchgrass under the stress of Pb in our study. Thus, the expressions of housekeeping genes are not universally stable among various species in response to different stress conditions. The selection of internal reference genes should be expanded beyond the housekeeping gene families. Cyclophilins (CyPs) are ubiquitous proteins functioning in the folding of certain proteins involved in signal transduction processes. In *Solanum tuberosum* L., the level of a cyclophilin gene mRNA accumulation is stimulated by the application of abscisic acid and methyl jasmonate in tubers. However, treatment with fungal elicitor or salicilic acid has no such obvious effect [43]. Moreover, *CYP* mRNA synthesis was also shown to be variable in maize and bean with mercuric chloride treatment and in other abiotic stresses conditions, such as heat, wounding, salt stress, and low temperature [44,45]. In addition, different drugs significantly induced *CYP* transcription in human tissues [46]. In this study, *CYP2* was found to be unstable as a reference gene in all samples except for root tissues treated with Pb. Therefore, particular caution should be taken when *CYP* is considered as a reliable reference gene.

To validate the selected internal reference genes from the screen, we chose *PvZIP4* and *PvPDR8* encoding the metal transporters as the target genes with expression induced by heavy metal stress [20,22]. The expression of these target genes in response to Cd and Pb stress was analyzed using two optimal and one poorly stable reference genes. Our results demonstrated that the target genes exhibited a general expression pattern in response to heavy metal stress when *CYP5/UBQ1*, *U2AF/ACT12*, *eEF1a/U2AF,* and *18S rRNA/ACT12* were used as the internal reference genes, while irregular patterns were shown with *CYP2* selected to be the reference gene for RT-qPCR analysis. It suggested that the selection of proper internal reference genes is essential for RT-qPCR quantitative analysis.

## 5. Conclusions

Our study provides a good reference for selecting proper internal reference genes to study the expression of target genes under heavy metal stress in switchgrass. According to the results of RefFinder, *U2AF* was the best reference gene in Cd, Hg, Cr, and As treated leaves as well as in Hg, Pb, As, and Cr stressed root tissues. *18S rRNA* was considered the most stable reference gene in Pb treated leaf tissues. *CYP5* was determined to be the optimal reference gene in Cd treated root tissues. While the least stable reference gene was identified to be *CYP2* in all tested samples except for root tissues stressed by Pb. In addition, the choice of the combination of the appropriate internal reference genes can significantly impact the analysis of the target gene expression pattern in response to different heavy metal stresses. Our findings established a solid foundation to further study the gene regulatory network of switchgrass in response to heavy metal stress.

## Figures and Tables

**Table 1:** Expression stability values for switchgrass candidate reference genes calculated by BestKeeper.

Rank	Total	CdL	CdR	PbL	PbR	HgL	HgR	CrL	CrR	AsL	AsR
1	*U2AF* (2.51 ± 0.61)	*UCE* (1.09 ± 0.24)	*eEF4a* (1.16 ± 0.33)	*U2AF* (1.01 ± 0.24)	*UBC* (1.10 ± 0.24)	*U2AF* (1.07 ± 0.25)	*U2AF* (2.54 ± 0.65)	*UBC* (1.26 ± 0.27)	*FTSH4* (0.72 ± 0.18)	*U2AF* (1.40 ± 0.34)	*eEF4a* (0.87 ± 0.26)
2	*eEF4a* (2.54 ± 0.74)	*FTSH4* (1.28 ± 0.3)	*ACT12* (1.37 ± 0.28	*ACT12* (1.10 ± 0.22)	*eEF1a* (1.14 ± 0.23)	*UCE* (1.26 ± 0.27)	*ACT2* (2.59 ± 0.78)	*U2AF* (1.29 ± 0.31)	*UCE* (1.10 ± 0.25)	*UBQ2* (1.46 ± 0.28)	*ACT2* (1.02 ± 0.31)
3	*ACT12* (2.67 ± 0.55)	*U2AF* (1.41 ± 0.33)	*U2AF* (1.42 ± 0.34)	*FTSH4* (1.31 ± 0.31)	*UCE* (1.49 ± 0.32)	*FTSH4* (1.34 ± 0.31)	*UBC* (2.73 ± 0.66)	*ACT12* (1.33 ± 0.27)	*UBC* (1.16 ± 0.27)	*UCE* (1.65 ± 0.37)	*U2AF* (1.05 ± 0.25)
4	*FTSH4* (3.24 ± 0.78)	*ACT12* (1.59 ± 0.32)	*UBQ1* (1.60 ± 0.32)	*UBQ1* (1.38 ± 0.29)	*eEF4a* (1.50 ± 0.43)	*ACT12* (1.34 ± 0.28)	*UBQ6* (3.00 ± 0.68)	*UCE* (1.57 ± 0.33)	*ACT12* (1.40 ± 0.29)	*eEF1a* (1.78 ± 0.40)	*UBQ2* (1.07 ± 0.20)
5	*UBQ2* (3.41 ± 0.62)	*UBC* (1.86 ± 0.38)	*CYP5* (1.62 ± 0.34)	*18S rRNA* (1.45 ± 0.13)	*U2AF* (1.54 ± 0.37)	*UBQ1* (1.41 ± 0.30)	*eEF4a* (3.27 ± 1.00)	*eEF4a* (1.97 ± 0.57)	*eEF4a* (1.55 ± 0.46)	*ACT12* (1.84 ± 0.38)	*eEF1a* (1.20 ± 0.27)
6	*CYP5* (3.59 ± 0.78)	*UBQ2* (2.19 ± 0.41)	*FTSH4* (1.71 ± 0.40)	*UCE* (1.53 ± 0.32)	*UBQ2* (2.00 ± 0.35)	*eEF4a* (1.68 ± 0.48)	*ACT12* (3.32 ± 0.70)	*FTSH4* (2.11 ± 0.49)	*U2AF* (1.61 ± 0.41)	*UBC* (1.97 ± 0.43)	*CYP5* (1.43 ± 0.32)
7	*UCE* (3.82 ± 0.83)	*eEF4a* (2.23 ± 0.65)	*eEF1a* (1.82 ± 0.38)	*eEF4a* (1.66 ± 0.47)	*UBQ1* (2.07 ± 0.41)	*UBC* (1.89 ± 0.40)	*UCE* (3.96 ± 0.89)	*CYP5* (3.25 ± 0.70)	*eEF1a* (1.89 ± 0.41)	*eEF4a* (2.11 ± 0.62)	*FTSH4* (1.46 ± 0.35)
8	*UBQ1* (3.87 ± 0.82)	*CYP5* (2.60 ± 0.57)	*UCE* (2.12 ± 0.45)	*CYP5* (1.68 ± 0.36)	*CYP5* (2.18 ± 0.45)	*UBQ2* (2.06 ± 0.39)	*FTSH4* (4.04 ± 1.08)	*UBQ2* (3.27 ± 0.60)	*ACT2* (2.27 ± 0.71)	*FTSH4* (2.22 ± 0.53)	*UBQ1* (1.61 ± 0.35)
9	*eEF1a* (4.12 ± 0.88)	*UBQ1* (2.87 ± 0.62)	*UBC* (2.18 ± 0.48)	*UBC* (1.91 ± 0.40)	*FTSH4* (2.28 ± 0.55)	*UBQ6* (2.10 ± 0.46)	*CYP5* (4.13 ± 0.96)	*UBQ6* (3.91 ± 0.89)	*CYP5* (2.63 ± 0.60)	*CYP5* (2.57 ± 0.55)	*UBQ6* (1.74 ± 0.38)
10	*UBC* (4.28 ± 0.93)	*eEF1a* (3.13 ± 0.66)	*UBQ2* (2.48 ± 0.42)	*UBQ2* (2.17 ± 0.40)	*ACT2* (2.69 ± 0.74)	*CYP5* (2.29 ± 0.49)	*UBQ2* (4.18 ± 0.77)	*UBQ1* (3.98 ± 0.85)	*UBQ6* (2.96 ± 0.65)	*UBQ1* (2.60 ± 0.57)	*UCE* (2.01 ± 0.48)
11	*ACT2* (4.35 ± 1.29)	*ACT2* (3.3 ± 0.98)	*ACT2* (2.78 ± 0.82)	*eEF1a* (2.59 ± 0.54)	*UBQ6* (2.91 ± 0.58)	*18S rRNA* (2.63 ± 0.25)	*eEF1a* (4.26 ± 0.99)	*eEF1a* (4.36 ± 0.92)	*UBQ1* (2.98 ± 0.66)	*UBQ6* (3.43 ± 0.76)	*ACT12* (2.23 ± 0.45)
12	*UBQ6* (4.38 ± 0.95)	*18S rRNA* (3.86 ± 0.38)	*UBQ6* (3.06 ± 0.62)	*UBQ6* (2.96 ± 0.65)	*CYP2* (3.72 ± 0.91)	*eEF1a* (2.96 ± 0.62)	*UBQ1* (4.76 ± 1.06)	*18S rRNA* (4.93 ± 0.48)	*UBQ2* (3.05 ± 0.54)	*18S rRNA* (3.97 ± 0.42)	*UBC* (2.57 ± 0.54)
13	*18S rRNA* (7.61 ± 0.78)	*UBQ6* (4.29 ± 0.95)	*18S rRNA* (4.75 ± 0.51)	*ACT2* (3.35 ± 0.96)	*ACT12* (4.43 ± 0.88)	*ACT2* (4.01 ± 1.20)	*CYP2* (5.01 ± 1.34)	*ACT2* (5.38 ± 1.57)	*CYP2* (3.89 ± 1.00)	*ACT2* (4.78 ± 1.44)	*18S rRNA* (3.76 ± 0.42)
14	*CYP2* (11.27 ± 3.11)	*CYP2* (6.36 ± 1.91)	*CYP2* (5.32 ± 1.33)	*CYP2* (10.46 ± 3.09)	*18S rRNA* (7.41 ± 0.75)	*CYP2* (14.04± 3.97)	*18S rRNA* (9.21 ± 1.05)	*CYP2* (10.69 ± 3.06)	*18S rRNA* (5.76 ± 0.65)	*CYP2* (8.34 ± 2.50)	*CYP2* (6.52 ± 1.75)

**Table 2:** Expression stability values for switchgrass candidate reference genes calculated by NormFinder.

Rank	Total	CdL	CdR	PbL	PbR	HgL	HgR	CrL	CrR	AsL	AsR
1	*U2AF* (0.463)	*U2AF* (0.095)	*CYP5* (0.138)	*18S rRNA* (0.105)	*eEF1a* (0.175)	*ACT12* (0.130)	*U2AF* (0.236)	*ACT12* (0.236)	*U2AF* (0.081)	*UBQ2* (0.192)	*U2AF* (0.079)
2	*CYP5* (0.484)	*ACT12* (0.269)	*FTSH4* (0.182)	*ACT12* (0.224)	*UCE* (0.190)	*CYP5* (0.159)	*UBC* (0.247)	*UBC* (0.313)	*UCE* (0.179)	*ACT12* (0.302)	*CYP5* (0.138)
3	*UBQ1* (0.539)	*UBQ1* (0.279)	*U2AF* (0.184)	*U2AF* (0.229)	*UBC* (0.247)	*UBQ1* (0.212)	*UBQ2* (0.251)	*U2AF* (0.369)	eEF4a (0.294)	*U2AF* (0.326)	*UBQ6* (0.273)
4	*eEF4a* (0.569)	18S rRNA (0.384)	*UBQ1* (0.201)	*FTSH4* (0.276)	*U2AF* (0.344)	*UCE* (0.274)	*UCE* (0.258)	*UCE* (0.375)	*FTSH4* (0.299)	*18S rRNA* (0.346)	*UBQ2* (0.275)
5	*eEF1a* (0.603)	*UCE* (0.414)	*UBQ2* (0.241)	*UCE* (0.279)	*UBQ1* (0.364)	*UBQ6* (0.293)	*eEF1a* (0.329)	*FTSH4* (0.548)	*eEF1a* (0.311)	*FTSH4* (0.398)	*eEF4a* (0.302)
6	*UCE* (0.728)	*FTSH4* (0.426)	*UCE* (0.288)	*UBC* (0.309)	*UBQ2* (0.483)	*UBQ2* (0.307)	*CYP5* (0.349)	*18S rRNA* (0.568)	*CYP5* (0.315)	*UBQ1* (0.403)	*UCE* (0.345)
7	*UBQ2* (0.748)	*CYP5* (0.437)	*UBC* (0.305)	*UBQ2* (0.316)	*CYP5* (0.513)	*U2AF* (0.320)	*18S rRNA* (0.386)	*eEF4a* (0.655)	*UBC* (0.366)	*eEF1a* (0.425)	*UBQ1* (0.347)
8	*ACT12* (0.757)	*eEF1a* (0.451)	*UBQ6* (0.397)	*UBQ1* (0.346)	*eEF4a* (0.546)	*18S rRNA* (0.346)	*eEF4a* (0.428)	*UBQ2* (0.743)	*UBQ2* (0.390)	*UBC* (0.476)	*UBC* (0.359)
9	*18S rRNA* (0.812)	*UBQ2* (0.494)	*eEF1a* (0.404)	*CYP5* (0.420)	*UBQ6* (0.633)	*eEF1a* (0.443)	*UBQ1* (0.462)	*CYP5* (0.851)	*UBQ1* (0.503)	*CYP5* (0.509)	*eEF1a* (0.370)
10	*FTSH4* (0.869)	*eEF4a* (0.539)	*ACT12* (0.535)	*eEF4a* (0.548)	*FTSH4* (0.686)	*eEF4a* (0.510)	*UBQ6* (0.625)	*UBQ6* (1.084)	*UBQ6* (0.509)	*UCE* (0.596)	*ACT12* (0.520)
11	*UBQ6* (0.903)	*UBC* (0.586)	*eEF4a* (0.557)	*eEF1a* (0.555)	*ACT2* (0.832)	*FTSH4* (0.576)	*FTSH4* (0.653)	*UBQ1* (1.162)	*ACT12* (0.521)	*eEF4a* (0.642)	*ACT2* (0.541)
12	*UBC* (1.038)	*UBQ6* (1.082)	*18S rRNA* (0.716)	*UBQ6* (0.699)	*18S rRNA* (0.877)	*UBC* (0.627)	*CYP2* (0.972)	*eEF1a* (1.247)	*18S rRNA* (0.601)	*UBQ6* (0.787)	*FTSH4* (0.622)
13	*ACT2* (1.467)	*ACT2* (1.273)	*ACT2* (1.016)	*ACT2* (1.249)	*CYP2* (1.566)	*ACT2* (1.615)	*ACT12* (1.338)	*ACT2* (1.840)	*ACT2* (1.093)	*ACT2* (1.825)	*18S rRNA* (0.732)
14	*CYP2* (3.391)	*CYP2* (2.651)	*CYP2* (1.825)	*CYP2* (3.522)	*ACT12* (1.580)	*CYP2* (4.373)	*ACT2* (1.456)	*CYP2* (3.497)	*CYP2* (1.147)	*CYP2* (3.037)	*CYP2* (2.221)

**Table 3:** The most stable and least stable combination of reference genes based on RefFinder analysis.

Method	Stability (High-Low)													
	1	2	3	4	5	6	7	8	9	10	11	12	13	14
	**Cd L**													
BestKeeper	*UCE*	*FTSH4*	*ACT12*	*U2AF*	*18Sr*	*UBC*	*UBQ2*	*CYP5*	*UBQ1*	*eEF4a*	*eEF1a*	*UBQ6*	*ACT2*	*CYP2*
NormFinder	*U2AF*	*ACT12*	*UBQ1*	*18Sr*	*UCE*	*FTSH4*	*CYP5*	*eEF1a*	*UBQ2*	*eEF4a*	*UBC*	*UBQ6*	*ACT2*	*CYP2*
Genorm	*U2AF*	*ACT12*	*UCE*	*FTSH4*	*UBC*	*UBQ2*	*18Sr*	*CYP5*	*UBQ1*	*eEF1a*	*eEF4a*	*UBQ6*	*ACT2*	*CYP2*
RefFinder	*U2AF*	*ACT12*	*UCE*	*FTSH4*	*18Sr*	*UBQ1*	*UBQ2*	*CYP5*	*UBC*	*eEF1a*	*eEF4a*	*UBQ6*	*ACT2*	*CYP2*
	**Pb L**													
BestKeeper	*18Sr*	*ACT12*	*U2AF*	*UBQ1*	*FTSH4*	*UCE*	*CYP5*	*UBC*	*UBQ2*	*eEF4a*	*eEF1a*	*UBQ6*	*ACT2*	*CYP2*
NormFinder	*18Sr*	*ACT12*	*U2AF*	*FTSH4*	*UCE*	*UBC*	*UBQ2*	*UBQ1*	*CYP5*	*eEF4a*	*eEF1a*	*UBQ6*	*ACT2*	*CYP2*
Genorm	*18Sr*	*ACT12*	*FTSH4*	*U2AF*	*UCE*	*CYP5*	*UBQ1*	*UBC*	*UBQ2*	*eEF1a*	*eEF4a*	*UBQ6*	*ACT2*	*CYP2*
RefFinder	*18Sr*	*ACT12*	*U2AF*	*FTSH4*	*UCE*	*UBQ1*	*CYP5*	*UBC*	*UBQ2*	*eEF4a*	*eEF1a*	*UBQ6*	*ACT2*	*CYP2*
	**Hg L**													
BestKeeper	*18Sr*	*U2AF*	*UCE*	*ACT12*	*UBQ1*	*FTSH4*	*UBQ2*	*UBC*	*UBQ6*	*eEF4a*	*CYP5*	*eEF1a*	*ACT2*	*CYP2*
NormFinder	*ACT12*	*CYP5*	*UBQ1*	*UCE*	*UBQ6*	*UBQ2*	*U2AF*	*18Sr*	*eEF1a*	*eEF4a*	*FTSH4*	*UBC*	*ACT2*	*CYP2*
Genorm	*UBQ6*	*CYP5*	*U2AF*	*ACT12*	*UCE*	*18Sr*	*UBQ1*	*eEF4a*	*UBQ2*	*eEF1a*	*FTSH4*	*UBC*	*ACT2*	*CYP2*
RefFinder	*ACT12*	*U2AF*	*CYP5*	*UBQ6*	*UCE*	*UBQ1*	*18Sr*	*UBQ2*	*eEF4a*	*FTSH4*	*eEF1a*	*UBC*	*ACT2*	*CYP2*
	**Cr L**													
BestKeeper	*UBC*	*ACT12*	*U2AF*	*UCE*	*18Sr*	*FTSH4*	*eEF4a*	*UBQ2*	*CYP5*	*UBQ1*	*UBQ6*	*eEF1a*	*ACT2*	*CYP2*
NormFinder	*ACT12*	*UBC*	*U2AF*	*UCE*	*FTSH4*	*18Sr*	*eEF4a*	*UBQ2*	*CYP5*	*UBQ6*	*UBQ1*	*eEF1a*	*ACT2*	*CYP2*
Genorm	*UCE*	*UBC*	*U2AF*	*ACT12*	*FTSH4*	*18Sr*	*UBQ2*	*eEF4a*	*CYP5*	*UBQ1*	*eEF1a*	*UBQ6*	*ACT2*	*CYP2*
RefFinder	*UBC*	*U2AF*	*ACT12*	*UCE*	*FTSH4*	*18Sr*	*eEF4a*	*UBQ2*	*CYP5*	*UBQ1*	*UBQ6*	*eEF1a*	*ACT2*	*CYP2*
	**As L**													
BestKeeper	*UBQ2*	*U2AF*	*UCE*	*ACT12*	*eEF1a*	*18Sr*	*UBC*	*FTSH4*	*CYP5*	*UBQ1*	*eEF4a*	*UBQ6*	*ACT2*	*CYP2*
NormFinder	*UBQ2*	*ACT12*	*U2AF*	*18Sr*	*FTSH4*	*UBQ1*	*eEF1a*	*UBC*	*CYP5*	*UCE*	*eEF4a*	*UBQ6*	*ACT2*	*CYP2*
Genorm	*U2AF*	*ACT12*	*UBQ2*	*18Sr*	*CYP5*	*eEF1a*	*UBQ1*	*FTSH4*	*eEF4a*	*UCE*	*UBC*	*UBQ6*	*ACT2*	*CYP2*
RefFinder	*UBQ2*	*U2AF*	*ACT12*	*18Sr*	*eEF1a*	*UBQ1*	*FTSH4*	*CYP5*	*UCE*	*UBC*	*eEF4a*	*UBQ6*	*ACT2*	*CYP2*
	**Cd R**													
BestKeeper	*ACT12*	*UBQ1*	*eEF4a*	*U2AF*	*CYP5*	*eEF1a*	*FTSH4*	*UBQ2*	*UCE*	*UBC*	*18Sr*	*UBQ6*	*ACT2*	*CYP2*
NormFinder	*CYP5*	*FTSH4*	*U2AF*	*UBQ1*	*UBQ2*	*UCE*	*UBC*	*UBQ6*	*eEF1a*	*ACT12*	*eEF4a*	*18Sr*	*ACT2*	*CYP2*
Genorm	*UBQ1*	*FTSH4*	*CYP5*	*UBQ2*	*U2AF*	*UCE*	*UBC*	*UBQ6*	*eEF1a*	*eEF4a*	*ACT12*	*18Sr*	*ACT2*	*CYP2*
RefFinder	*CYP5*	*UBQ1*	*FTSH4*	*U2AF*	*UBQ2*	*ACT12*	*UCE*	*UBC*	*eEF4a*	*eEF1a*	*UBQ6*	*18Sr*	*ACT2*	*CYP2*
	**Pb R**													
BestKeeper	*eEF1a*	*UBC*	*UCE*	*UBQ2*	*U2AF*	*UBQ1*	*eEF4a*	*CYP5*	*FTSH4*	*UBQ6*	*ACT2*	*18Sr*	*ACT12*	*CYP2*
NormFinder	*eEF1a*	*UCE*	*UBC*	*U2AF*	*UBQ1*	*UBQ2*	*CYP5*	*eEF4a*	*UBQ6*	*FTSH4*	*ACT2*	*18Sr*	*CYP2*	*ACT12*
Genorm	*eEF1a*	*U2AF*	*UCE*	*UBQ1*	*CYP5*	*UBC*	*UBQ6*	*UBQ2*	*eEF4a*	*FTSH4*	*18Sr*	*ACT2*	*CYP2*	*ACT12*
RefFinder	*eEF1a*	*U2AF*	*UCE*	*UBC*	*UBQ1*	*UBQ2*	*CYP5*	*eEF4a*	*UBQ6*	*FTSH4*	*18Sr*	*ACT2*	*CYP2*	*ACT12*
	**Hg R**													
BestKeeper	*U2AF*	*UBC*	*UBQ6*	*ACT12*	*UBQ2*	*ACT2*	*UCE*	*CYP5*	*eEF1a*	*eEF4a*	*18Sr*	*UBQ1*	*FTSH4*	*CYP2*
NormFinder	*U2AF*	*UBC*	*UBQ2*	*UCE*	*eEF1a*	*CYP5*	*18Sr*	*eEF4a*	*UBQ1*	*UBQ6*	*FTSH4*	*CYP2*	*ACT12*	*ACT2*
Genorm	*UBQ1*	*UCE*	*eEF1a*	*eEF4a*	*CYP5*	*18Sr*	*UBC*	*U2AF*	*UBQ2*	*FTSH4*	*UBQ6*	*CYP2*	*ACT12*	*ACT2*
RefFinder	*UCE*	*U2AF*	*UBC*	*eEF1a*	*CYP5*	*UBQ2*	*UBQ1*	*eEF4a*	*18Sr*	*UBQ6*	*ACT12*	*FTSH4*	*ACT2*	*CYP2*
	**Cr R**													
BestKeeper	*FTSH4*	*UCE*	*UBC*	*ACT12*	*U2AF*	*eEF1a*	*eEF4a*	*UBQ2*	*CYP5*	*18Sr*	*UBQ6*	*UBQ1*	*ACT2*	*CYP2*
NormFinder	*U2AF*	*UCE*	*eEF4a*	*FTSH4*	*eEF1a*	*CYP5*	*UBC*	*UBQ2*	*UBQ1*	*UBQ6*	*ACT12*	*18Sr*	*ACT2*	*CYP2*
Genorm	*UCE*	*UBC*	*FTSH4*	*U2AF*	*eEF1a*	*CYP5*	*eEF4a*	*UBQ2*	*UBQ6*	*UBQ1*	*ACT12*	*18Sr*	*ACT2*	*CYP2*
RefFinder	*UCE*	*U2AF*	*FTSH4*	*UBC*	*eEF4a*	*eEF1a*	*CYP5*	*UBQ2*	*ACT12*	*UBQ1*	*UBQ6*	*18Sr*	*ACT2*	*CYP2*
	**As R**													
BestKeeper	*UBQ2*	*U2AF*	*eEF4a*	*eEF1a*	*ACT2*	*CYP5*	*UBQ1*	*FTSH4*	*UBQ6*	*18Sr*	*ACT12*	*UCE*	*UBC*	*CYP2*
NormFinder	*U2AF*	*CYP5*	*UBQ6*	*UBQ2*	*eEF4a*	*UCE*	*UBQ1*	*UBC*	*eEF1a*	*ACT12*	*ACT2*	*FTSH4*	*18Sr*	*CYP2*
Genorm	*eEF1a*	*eEF4a*	*UBQ1*	*UBQ6*	*CYP5*	*U2AF*	*UBQ2*	*ACT12*	*UBC*	*UCE*	*ACT2*	*FTSH4*	*18Sr*	*CYP2*
RefFinder	*U2AF*	*eEF4a*	*UBQ2*	*eEF1a*	*CYP5*	*UBQ6*	*UBQ1*	*UCE*	*ACT2*	*UBC*	*ACT12*	*FTSH4*	*18Sr*	*CYP2*
	**Total**													
BestKeeper	*ACT12*	*U2AF*	*UBQ2*	*eEF4a*	*FTSH4*	*CYP5*	*18Sr*	*UBQ1*	*UCE*	*eEF1a*	*UBC*	*UBQ6*	*ACT2*	*CYP2*
NormFinder	*U2AF*	*CYP5*	*UBQ1*	*eEF4a*	*eEF1a*	*UCE*	*UBQ2*	*ACT12*	*18Sr*	*FTSH4*	*UBQ6*	*UBC*	*ACT2*	*CYP2*
Genorm	*eEF4a*	*U2AF*	*CYP5*	*18Sr*	*FTSH4*	*eEF1a*	*UCE*	*UBQ1*	*UBC*	*ACT12*	*UBQ2*	*UBQ6*	*ACT2*	*CYP2*
RefFinder	*U2AF*	*eEF4a*	*CYP5*	*ACT12*	*UBQ1*	*eEF1a*	*18Sr*	*FTSH4*	*UCE*	*UBQ2*	*UBC*	*UBQ6*	*ACT2*	*CYP2*

## Supplementary Materials

The following are available online at https://www.mdpi.com/2073-4425/11/5/502/s1, Figure S1: Melting curves for fourteen candidate reference genes. Table S1. Reference genes and primer sequences. Table S2. Ct value for fourteen candidate reference genes under different heavy metal stresses.
