# Supplementary material for: Identification and Validation of Reference Genes for RT-qPCR Analysis in Switchgrass under Heavy Metal Stresses
Source: Genes (Basel). 2020 May 3;11(5):502. doi: 10.3390/genes11050502 (PMC7291066; doi:10.3390/genes11050502)
Supplement: Supplementary file 1 [file genes-11-00502-s001.pdf]

Supplementary Materials

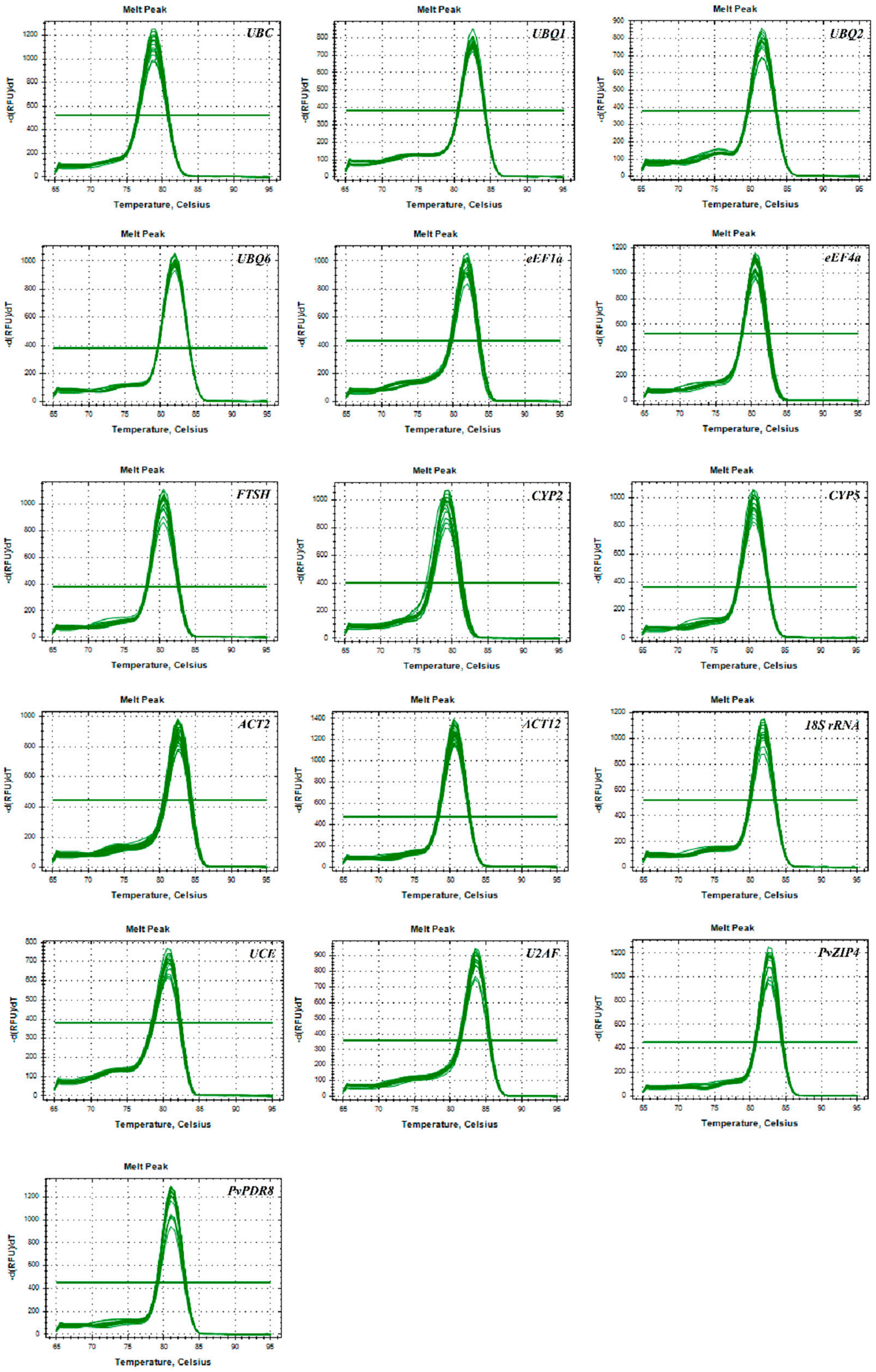

Figure S1. Melting curves for fourteen candidate reference genes.

Table S1. Reference genes and primer sequences.

Gene name	Gene description	GeneBank accession	5'-Primer Sequences (Forward/Reverse)-3'	Amplicon Length (bp)
18S rRNA	18S ribosomal RNA1	GR878775	CTACCACATCCAAGGAAG/CAATTACCAGACACTAACG	105
ACT12	Actin 12	GR878265	CAGCCATCCATGATCGGTATG/TGCCGTACAGGTCCTTTCTGA	100
ACT2	Actin 2	FL724919	GCGAGCTTCCCTGTAGGTA/CGAACCCAGCCTTCACCATAC	93
CYP2	Cyclophilin 2	FL942644	CTCAGACCACCTCTCAGA/CGTTCCTCCTTCGTTCAT	78
CYP5	Cyclophilin 5	FE633090	CACTACAAGGGAAGCACATTCCA/TTCACCACCCCTTCCATCAC	90
eEF1a	Eukariotic elongation factor 1-a	GR876801	CGGTTGGTCGTGTGGAGACT/TGGTGCATCTCAACAGACTTCAC	100
eEF4a	Eukaryotic initiation factor 4a	GR877213	TGATGTCATTCAGCAAGCACAA/GGCATTCAACCAGGCCATAG	95
FTSH4	Ftsh protease 4	FL791612	TGGATGGCTTTAAGCAGAATGA/CAAAACGCCCAGGTCTGACT	95
U2AF	Splicing factor U2af	FL907910	GGGTCAACTGCCCTTTTTACTTC/AGCACAAGAGTCGGCGATATG	100
UBC	Ubiquitin-conjugating enzyme	GR879761	ATCAACAGCAATGGAAGTATATG/GGAGAGCAGGACCTTAGA	84
UBQ1	Ubiquitin 1	FL955474	CAGCGAGGGCTCAATAATTCCA/TCTGGCGGACTACAATATCCA	82
UBQ2	Ubiquitin 2	FL920273.1	TTCGTGGTGGCCAGTAAG/AGAGACCAGAAGACCCAGGTACAG	83
UBQ6	Ubiquitin 6	FE609298	AGAAGCGCAAGAAGAAGACG/CCACCTTGTAGAACTGGAGCA	93
UCE	Ubiquitin-conjugating enzyme 2	GR879053	TATATGACGGAGGCTACT/CAGGTGGATGAAGAATAGA	147
PvZIP4	Zinc/iron regulated transporter protein 4	Pavir.J08901	GTGGTTCCATAGGTGAGATGTT/TAGAGGACCAGCCACAGTAA	163
PvPDR8	Pleiotropic drug resistance 8	PTHR19241	CTCTTCTCGGGCTTCTTCATTC/ACCTCTTCCAGGTCTCCATATT	128

Table S2. Ct value for fourteen candidate reference genes under different heavy metal stresses.

Cd Leaf

UBQ1	*UBQ2*	*UBQ6*	*FTSH4*	*CYP2*	*UCE*	*eEF1a*	*eEF4a*	*CYP5*	*18Sr*	*U2AF*	*UBC*	*ACT2*	*ACT12*
22.31	18.41	22.61	24.38	31.6	21.83	22.31	30.35	21.93	10.64	24.27	21.02	28.97	20.25
22.4	18.91	23.83	23.88	32.87	21.65	21.38	29.88	22.58	10.41	24	20.4	29.08	20.01
21.63	19.26	22.06	23.74	31.34	21.62	21.36	29.56	21.73	10.18	24.02	20.44	27.46	20.25
20.88	18.2	23.25	23.13	29.85	20.9	20.17	29.43	20.57	10.09	23.13	19.83	29.54	19.25
22.6	18.92	22.55	23.78	31.95	22.07	22.1	29.99	22.56	9.6	23.94	20.85	31.13	20.36
21.58	19.15	21.43	23.27	30.12	21.86	20.65	28.81	21.42	9.49	23.65	21.06	31.05	20.22
21	17.83	21.23	23.5	31.12	21.48	20.37	28.21	21.38	9.3	23.38	20.28	31.57	19.87
20.79	18.56	20.67	23.86	23.63	21.85	20.71	28.85	21.63	9.69	23.43	21.07	30.32	19.69
20.16	18.24	22.65	23.54	30.48	21.27	20.06	27.94	20.81	9.45	23.25	20.7	28.77	19.26
21.67	19.48	20.53	24.15	25.34	22.09	21.23	29.04	22.5	9.6	24.05	21.16	29.17	20.41
21.09	18.55	24.1	23.59	31.16	21.71	20.08	27.75	20.81	9.11	23.23	20	27.44	19.83
22.23	18.44	22.35	24.16	31.15	21.75	22.39	30.42	22.09	10.33	24.15	20.89	29.34	20.31
22.36	19.02	23.69	23.63	32.24	21.66	21.07	29.77	22.89	10.36	24.03	20.31	31.15	20.01
21.44	19.15	22.86	23.66	30.89	21.53	21.12	29.47	21.97	10.32	23.89	20.32	30.31	20.23
20.96	18.24	21.68	22.91	29.73	20.99	20.2	29.29	20.76	10.11	23.13	19.86	31.1	19.29
22.56	19.04	21.71	23.39	32.32	21.92	21.84	30.12	22.64	9.76	23.99	20.95	29.64	20.4
21.62	19.18	20.87	23.12	30.31	21.77	20.47	28.82	21.66	9.55	23.53	21.06	29.41	20.25
20.74	17.94	23.1	23.09	31.29	21.49	20.15	28.15	21.23	9.38	23.55	20.39	28.61	19.84
20.66	18.62	20.54	23.57	23.54	21.81	20.42	29.13	21.6	9.84	23.39	20.98	28.75	19.72

Pb Leaf

UBQ1	*UBQ2*	*UBQ6*	*FTSH4*	*CYP2*	*UCE*	*eEF1a*	*eEF4a*	*CYP5*	*18Sr*	*U2AF*	*UBC*	*ACT2*	*ACT12*
21.09	18.03	23.05	23.97	25.77	21.31	21.23	28.56	22.01	9.25	24.22	21.02	27.71	20.33
20.15	18.22	21.19	23.28	31.09	20.88	20.21	28.29	20.27	8.92	23.54	20.18	27.13	19.75
21.19	18.26	22.6	23.53	32.19	20.97	21.62	29.08	21.03	9.12	23.94	20.62	27.11	19.78
21.45	17.54	23.21	23.05	23.81	20.47	21.21	28.69	21.18	9.14	23.57	20.16	29.66	19.76
20.75	18.64	22.76	23.4	31.25	21.24	21.84	28.95	21.05	9.42	23.92	21.07	30.92	20.15
21.31	18.46	21.66	23.76	31.4	21.49	20.52	28.44	21.58	9.07	23.7	21.41	28.72	20.24
21.07	18.77	22.3	23.15	31.62	21.22	20.43	28.22	20.84	9.32	23.79	21.19	30.22	19.84
20.83	18	21.65	22.76	31.52	21.03	20.36	27.5	20.61	8.99	23.22	21.14	28.13	19.78
20.83	18.46	21.27	23.42	23.59	21.17	21.35	27.51	20.99	9.2	23.23	20.98	28.72	20.06
21.38	19.52	22.49	23.87	32.54	21.61	21.67	27.69	21.54	9.38	23.55	21.88	28.71	20.15
21.05	18.03	22.9	23.96	25.52	22.26	21.26	28.96	22.01	9.44	23.94	21.06	27.96	20.4
20.25	18.3	20.83	23.27	31.03	20.89	20.05	28.38	20.28	9.14	23.45	20.2	27.43	19.68
21.24	18.2	22.39	23.53	32.08	20.89	21.73	29.02	21.06	9.1	23.65	20.36	26.87	19.74
21.48	17.62	22.96	22.88	23.91	20.47	21.19	28.58	21.28	9.12	23.31	20.2	29.22	19.88
20.84	18.81	22.17	23.23	31.32	21.12	21.66	28.92	21.14	9.37	23.48	20.95	31.82	20.29
21.17	18.37	21.55	23.52	31.78	21.5	20.56	28.25	21.52	9.13	23.67	21.44	28.24	20.34
20.82	19.04	21.46	23.09	31.68	20.76	20.16	28.13	21.03	9.05	23.5	21.2	29.48	19.88
20.71	18.06	21.2	22.75	31.45	20.82	20.16	27.51	20.65	9.08	23.03	21.18	28.28	19.85
20.83	18.55	21	23.19	23.58	20.77	21.14	27.44	21.04	8.99	23.05	20.85	28.61	20.14
21.36	19.55	22.12	23.81	32.97	21.48	21.35	27.65	21.44	9.41	23.39	21.66	29.26	20.26

Hg Leaf

UBQ1	*UBQ2*	*UBQ6*	*FTSH4*	*CYP2*	*UCE*	*eEF1a*	*eEF4a*	*CYP5*	*18Sr*	*U2AF*	*UBC*	*ACT2*	*ACT12*
21.79	18.24	22.8	23.82	24.8	21.17	21.95	29.5	22.16	10.09	24.39	20.74	28.15	20.58
21.49	18.33	21.54	23.5	31.57	21.22	20.57	28.77	21.28	10	23.34	20.81	28.91	20.25
21.69	18.49	21.75	23.44	31.12	21	20.82	29.35	21.25	9.66	23.7	20.52	28.74	20.57
21.64	19.11	22.24	23.16	31.02	21.85	20.83	29.22	22.13	9.58	23.9	21.31	32.09	21.36
20.21	17.84	20.72	22.36	22.44	20.95	19.51	27.91	20.39	9.09	23.21	21.39	30.51	20.22
21.6	18.79	22.25	23.74	32.76	21.34	21.48	28.83	21.77	9.49	23.65	21.42	30.13	20.5
21.33	18.91	21.13	23.27	21.77	21.08	20.3	28.71	20.58	9.28	23.36	21.46	31.81	20.15
21.92	18.67	21.72	23.98	32.3	20.5	21.63	28.37	21.16	9.5	23.39	20.97	30.45	20.1
21.69	18.99	22.1	23.51	24.7	21.55	21.5	29.11	22.06	9.47	23.52	21.94	31.2	20.69
21.89	19.72	21.82	23.81	28.27	21.53	21.53	27.93	21.39	9.33	23.59	22	30.15	20.74
21.57	18.18	22.95	23.57	24.88	21.17	21.95	28.97	21.85	10.09	24.51	20.94	27.87	20.71
21.4	18.27	21.5	23.35	31.96	21.26	20.37	29.19	21.27	10.07	23.42	20.66	27.63	20.39
21.52	18.42	21.73	23.17	31.27	20.88	20.66	28.94	21.11	9.61	23.8	20.55	28.67	20.41
21.55	19.15	22.31	22.3	31.53	21.6	21.04	29.16	21.89	9.58	23.77	21.45	32.27	21.31
20.06	18.01	20.68	23.68	22.05	20.74	19.51	27.67	20.16	8.95	23.2	21.36	30.55	20.14
21.51	18.75	22.19	23.2	32.52	21.07	21.41	28.41	21.77	9.44	23.75	21.38	29.77	20.71
21.18	18.95	21.19	23.85	21.41	21.03	20.34	28.47	20.42	9.16	23.38	21.51	31.63	20.26
21.74	18.69	21.61	23.31	32.34	20.47	21.53	28.02	21.05	9.52	23.34	21.04	29.57	20.19
21.41	18.85	22.14	23.73	24.47	21.24	21.67	28.61	21.95	9.54	23.55	21.99	31.73	20.26
21.55	19.64	21.67	23.4	32.38	21.44	21.67	27.48	21.2	9.02	23.63	22.2	29.04	20.68

Cr Leaf

UBQ1	*UBQ2*	*UBQ6*	*FTSH4*	*CYP2*	*UCE*	*eEF1a*	*eEF4a*	*CYP5*	*18Sr*	*U2AF*	*UBC*	*ACT2*	*ACT12*
21.55	18.16	22.15	23.8	24.7	20.91	21.03	28.76	21.67	10.67	23.81	21.04	27.21	20.79
21.89	19.03	23.25	23.69	33.1	20.71	21.55	28.81	21.44	10.6	23.5	20.72	27.17	20.9
23.18	19.45	24.55	23.91	25.34	21.13	22.46	28.29	22.3	10.04	24.06	20.95	27.26	20.42
21.94	17.33	22.56	23	30.59	20.79	20.75	28.31	22.3	9.26	24.15	20.55	28.45	21.3
22.13	18.79	22.1	23.43	23.57	20.91	22.17	30.28	22.66	9.77	24.06	21.38	31.74	20.43
20.32	17.88	23.24	22.9	30.59	21.69	19.67	30.14	22.73	10.34	23.86	21.72	29.73	20.43
19.37	18.53	24.39	22.45	30.68	20.39	18.61	28.54	21.35	10	24.05	21.19	30.61	20.52
21.14	17.55	22.51	22.67	31.05	20.36	21.15	28.55	21.39	9.74	23.6	20.78	31.58	20.98
21.54	18.55	23.01	23.66	24.75	21.01	21.05	29.25	21.79	9.12	23.92	21.16	27.41	20.57
21.86	19.25	21.56	23.41	32.18	20.79	21.78	29.26	21.92	8.49	23.56	20.99	28	20.29
23.17	19.63	20.58	23.92	25.19	21.19	22.6	27.59	21.02	8.58	24.57	21.09	27.41	20.12
22.02	17.47	23.27	22.77	30.45	20.7	20.82	27.54	21.03	9.72	23.46	20.69	28.32	20.44
22.14	18.92	23.13	23.3	23.66	21.04	22.16	28.05	19.48	9.58	24.12	21.1	30.24	20.93
20.29	18.04	22.08	22.56	31.04	21.78	19.69	28.23	19.6	10.34	24.11	21.93	29.81	20.94
19.6	18.45	20.67	22.41	30.22	20.35	19	28.78	20.87	9.78	23.19	21.41	31.06	20.9
21.2	17.7	23.55	22.53	30.71	20.34	21.15	28.48	20.95	9.44	23.21	21.08	31.64	20.42

As Leaf

UBQ1	*UBQ2*	*UBQ6*	*FTSH4*	*CYP2*	*UCE*	*eEF1a*	*eEF4a*	*CYP5*	*18Sr*	*U2AF*	*UBC*	*ACT2*	*ACT12*
22.76	19.37	24.17	24.18	25.11	23.6	23.04	29.79	22.87	10.58	25.14	22.02	28.36	21.28
21.39	18.78	21.76	24.2	25.16	23.1	22.77	29.65	21.17	10.26	24.44	20.96	28.48	20.08
22.5	19.15	22.71	24.71	32.35	22.05	22.22	30.11	22.26	10.71	24.85	22.01	28.86	21.27
22.13	18.74	22.19	23.72	31.52	21.85	21.94	30	21.69	10.58	24.3	21.49	30.59	20.71
21.3	18.99	21.09	23.52	32.27	22.8	22.15	28.74	21.14	10.24	24.33	22.35	32.92	20.9
21.86	19.33	22.46	24.55	32.43	23.01	22.42	29.39	21.06	10.1	24.34	22.51	30.87	21.04
20.67	18.31	20.62	23.04	30.8	22.24	21.25	27.84	20.44	9.77	23.43	21.31	31.2	20.06
21.92	19.05	21.88	23.26	31.1	22.25	21.87	28.49	21.46	10.13	24.03	21.66	29.19	20.73
22.82	19.27	24.12	24.44	31.2	22.41	23.37	29.05	21.65	10.86	24.49	22.6	32.76	21.13
21.21	18.68	21.76	24.58	31.27	22.48	22.53	27.53	21.02	9.23	23.26	22.11	29.05	20.27
22.42	19.11	22.79	24.96	31.98	22.68	23.26	29.88	22.93	11.09	25.11	22.06	28.28	21.41
22.34	18.58	22.34	23.91	32.47	22.91	22.54	29.3	21.2	10.67	24.31	20.94	28.33	20.04
21.35	18.87	21.16	23.8	31.17	21.98	22.41	30.08	22.27	11.1	24.76	21.93	29.22	21.3
21.96	19.11	22.65	24.66	30.81	22.24	22.15	29.59	21.86	11.39	24.26	21.43	31.34	20.84
20.39	18.18	20.96	23.01	24.69	22.2	22.1	28.7	21.25	10.29	24.16	22.13	32.77	21.01
21.73	18.98	22.05	23.48	24.84	22.52	22.55	29.27	21.08	10.32	24.29	22.46	30.38	21.16

Cd Root

UBQ1	*UBQ2*	*UBQ6*	*FTSH4*	*CYP2*	*UCE*	*eEF1a*	*eEF4a*	*CYP5*	*18Sr*	*U2AF*	*UBC*	*ACT2*	*ACT12*
20.01	17.44	20.75	23.71	29.39	21.26	20.80	28.47	21.17	9.97	24.10	22.45	28.72	20.19
20.01	17.32	20.63	23.62	29.51	21.52	20.59	28.49	21.30	9.94	24.04	22.52	29.16	20.30
20.14	16.73	20.52	24.02	24.75	21.64	20.21	28.69	21.04	10.27	24.05	22.59	30.05	20.88
19.99	16.78	20.63	24.06	24.65	21.46	20.15	30.58	21.15	9.97	24.06	22.57	29.60	20.92
20.15	16.59	20.50	23.36	24.14	21.28	20.62	28.81	21.60	10.53	24.29	22.47	31.20	20.37
20.02	16.66	20.46	23.24	24.06	21.46	20.47	28.79	21.58	10.42	24.32	22.49	31.58	20.52
19.82	17.06	19.67	23.37	23.45	21.44	20.39	28.80	21.20	11.24	24.42	22.18	30.02	19.90
19.74	17.14	19.22	23.35	23.49	21.65	20.30	29.00	21.31	11.15	24.20	22.19	29.71	20.11
19.52	16.33	19.05	22.89	23.99	20.26	19.95	28.12	20.25	10.17	23.19	21.09	28.96	20.03
19.35	16.28	19.15	22.92	24.01	20.45	19.77	28.29	20.32	10.04	23.15	21.19	28.08	20.33
19.76	16.64	19.67	23.09	24.21	20.30	20.79	28.43	20.83	10.70	23.46	21.29	30.37	20.07
19.68	16.57	19.89	23.05	24.26	20.59	20.69	28.50	20.89	10.58	23.29	21.35	29.28	20.14
19.75	16.34	19.80	23.16	23.68	20.50	21.13	29.12	21.05	11.65	23.83	21.54	28.61	20.64
19.76	16.40	20.02	23.23	23.74	20.76	21.00	28.95	21.09	11.44	23.94	21.52	27.66	20.80
21.06	17.83	21.45	24.36	25.68	21.38	21.49	28.93	22.08	11.38	24.32	22.08	29.51	20.06
21.12	17.75	21.42	24.36	25.70	21.23	21.31	28.92	21.86	11.11	24.21	22.00	30.58	19.86

Pb Root

UBQ1	*UBQ2*	*UBQ6*	*FTSH4*	*CYP2*	*UCE*	*eEF1a*	*eEF4a*	*CYP5*	*18Sr*	*U2AF*	*UBC*	*ACT2*	*ACT12*
20.25	17.13	21.15	23.52	24.77	21.92	20.18	27.77	21.40	9.45	24.03	22.24	29.05	19.22
20.18	17.20	21.27	23.56	24.85	22.04	20.21	28.05	21.64	9.85	24.05	22.21	28.80	19.28
18.90	16.96	19.05	22.94	27.61	20.80	19.27	27.52	19.30	8.66	22.76	21.71	27.25	23.22
18.82	17.09	19.28	23.01	27.39	20.64	19.47	27.34	19.39	8.68	22.72	21.74	27.37	23.38
19.90	17.62	20.42	23.85	23.94	21.18	20.14	28.60	20.80	9.84	23.92	22.33	27.02	19.72
19.84	17.61	20.43	23.73	23.87	21.29	20.16	28.39	20.84	9.63	23.91	22.13	27.49	19.73
20.03	18.47	20.42	24.47	22.94	21.14	20.30	28.69	20.82	10.35	24.41	22.29	26.29	19.28
19.94	18.56	20.38	24.37	22.87	21.24	20.17	28.63	20.96	10.07	24.24	22.27	26.28	19.57
20.02	17.77	20.10	23.99	24.08	20.76	20.13	28.43	20.44	10.18	23.69	21.71	26.60	19.41
19.79	17.99	20.28	23.86	24.14	20.81	19.96	28.73	20.64	9.99	23.72	21.73	26.78	19.48
19.17	17.81	19.33	24.12	24.42	20.74	20.09	28.31	20.06	9.43	23.61	22.13	25.85	18.78
19.07	17.92	19.37	24.10	24.42	21.01	19.99	28.28	20.22	9.35	23.63	22.03	26.34	18.53
19.48	18.04	19.29	25.65	24.27	21.34	20.75	29.58	20.27	11.64	24.33	22.13	27.41	19.19
19.31	17.91	19.35	25.59	24.27	21.37	20.71	29.72	20.34	11.66	24.45	21.92	27.81	19.47
19.95	17.96	19.74	24.51	23.02	21.44	20.30	28.44	20.57	11.57	24.02	21.53	28.08	19.27
20.03	17.81	19.75	24.43	23.03	21.50	20.15	28.36	20.49	11.14	24.02	21.58	28.16	19.20

Hg Root

UBQ1	*UBQ2*	*UBQ6*	*FTSH4*	*CYP2*	*UCE*	*eEF1a*	*eEF4a*	*CYP5*	*18Sr*	*U2AF*	*UBC*	*ACT2*	*ACT12*
20.87	17.72	22.77	25.16	25.23	21.26	21.68	28.66	22.27	10.24	25.11	22.98	30.50	22.24
20.67	17.61	22.87	25.14	24.94	21.25	21.64	28.97	22.24	10.27	24.80	23.40	30.19	22.34
20.89	17.48	21.23	25.02	24.43	21.34	22.10	29.63	21.46	10.45	24.75	23.41	30.69	20.36
20.79	17.57	21.15	25.11	24.46	21.40	21.85	29.34	21.86	10.33	24.93	23.25	30.34	20.39
20.91	17.38	21.16	26.63	26.34	21.66	22.15	29.91	22.06	10.30	25.18	23.47	31.08	22.13
20.86	17.24	21.16	26.38	26.23	21.74	22.26	29.79	22.04	10.39	25.16	23.42	32.32	22.17
22.17	17.71	22.49	27.12	25.74	22.41	23.13	30.31	23.03	10.63	26.00	24.26	28.47	20.17
22.03	17.75	22.43	27.02	25.57	22.33	23.18	30.59	23.27	10.66	25.64	23.99	29.19	20.46
22.32	18.40	22.49	25.94	26.33	22.70	22.63	30.47	22.96	11.06	25.53	24.10	29.01	20.74
22.26	18.41	22.63	25.89	26.47	22.69	22.71	30.80	22.95	10.87	25.31	23.97	28.95	20.93
23.54	19.28	23.45	27.12	27.75	23.49	24.00	31.59	23.95	12.16	25.96	24.62	30.25	21.61
23.49	19.30	23.51	27.06	27.67	23.59	24.07	31.92	23.94	12.05	26.19	24.60	30.49	22.31
23.33	19.20	22.99	28.00	29.62	23.33	24.25	31.91	24.09	12.66	26.26	24.97	29.41	21.16
23.27	19.17	23.05	28.08	29.44	23.24	24.20	31.75	24.22	12.58	26.43	24.39	29.32	21.27
23.92	19.44	23.30	29.19	28.12	24.21	25.07	32.17	25.13	13.65	27.31	25.62	30.90	20.50
23.98	19.50	23.35	28.98	27.94	24.42	24.89	32.28	25.24	13.41	27.24	25.79	30.47	20.50

Cr Root

UBQ1	*UBQ2*	*UBQ6*	*FTSH4*	*CYP2*	*UCE*	*eEF1a*	*eEF4a*	*CYP5*	*18Sr*	*U2AF*	*UBC*	*ACT2*	*ACT12*
21.33	17.19	21.35	24.29	24.78	22.32	21.18	28.79	21.99	10.24	24.79	23.41	30.99	21.68
21.59	17.25	21.50	24.37	24.66	22.53	21.20	28.78	22.11	10.65	24.71	23.51	30.17	21.15
20.96	17.13	21.21	24.44	23.57	22.08	21.56	29.16	21.78	10.08	24.25	23.02	30.80	20.77
21.08	16.84	21.31	24.17	23.60	22.10	21.58	29.15	21.89	10.23	24.32	23.19	31.75	20.89
21.44	17.06	21.26	24.48	26.33	22.74	22.19	29.40	22.55	10.52	25.00	23.67	32.93	20.76
21.40	17.04	21.31	24.36	26.24	22.52	22.19	29.29	22.55	10.45	24.96	23.86	32.34	21.10
22.05	17.32	21.53	24.49	28.41	22.82	22.27	30.02	22.70	11.25	25.62	23.19	30.86	21.44
22.02	17.42	21.76	24.55	28.13	22.53	22.20	30.16	22.87	11.13	25.43	23.06	31.70	21.54
23.13	18.22	22.50	24.95	26.19	22.81	22.52	30.31	23.37	11.73	25.50	23.44	29.92	20.48
23.14	18.44	22.55	24.79	26.13	22.61	22.49	30.36	23.43	11.31	25.50	23.44	30.02	20.68
23.22	18.39	22.49	24.44	25.82	23.06	21.83	30.48	23.63	12.13	25.48	23.73	31.52	20.98
23.14	18.31	22.49	24.39	25.97	22.78	21.84	30.44	23.57	12.04	25.46	23.59	31.43	21.01
21.55	17.97	20.84	24.51	24.85	22.44	21.38	29.74	22.16	12.21	25.03	23.15	31.14	20.60
21.45	17.84	21.02	24.59	24.96	22.03	21.27	29.66	22.15	12.18	25.10	23.00	31.96	20.96
22.34	18.32	23.01	24.97	26.13	22.72	22.31	29.73	23.61	11.76	25.96	23.98	30.08	21.47
22.27	18.39	23.09	24.76	26.06	22.98	22.29	29.75	23.48	11.45	25.70	24.01	30.61	21.35

As Root

UBQ1	*UBQ2*	*UBQ6*	*FTSH4*	*CYP2*	*UCE*	*eEF1a*	*eEF4a*	*CYP5*	*18Sr*	*U2AF*	*UBC*	*ACT2*	*ACT12*
21.76	18.36	22.53	24.09	31.88	24.95	21.79	29.30	22.35	10.82	24.71	21.58	30.31	20.09
21.69	18.56	22.44	24.01	31.60	24.98	21.69	29.35	22.64	11.29	24.80	21.70	30.61	19.74
21.77	18.29	22.78	23.37	28.95	24.42	22.06	29.57	22.67	10.69	24.18	21.54	30.83	21.07
21.61	18.44	22.46	23.50	28.87	24.07	22.20	29.61	23.03	10.70	24.06	21.74	30.81	21.16
22.04	18.46	22.16	24.59	26.18	24.21	22.41	30.01	22.24	11.31	24.48	21.87	30.06	20.50
22.05	18.38	22.15	24.51	26.75	24.26	22.46	29.92	22.26	11.07	24.50	21.91	30.03	21.14
21.82	18.06	21.95	23.71	25.45	23.42	22.28	29.47	21.76	10.80	24.02	20.55	30.09	20.28
21.76	18.02	21.89	23.66	25.51	23.58	22.19	29.49	22.05	10.57	23.84	20.56	30.30	20.34
21.96	18.21	22.45	24.13	26.66	23.22	22.53	29.82	22.27	11.76	24.27	20.46	30.00	20.44
22.01	18.11	22.64	24.08	26.80	23.42	22.39	29.98	22.38	11.34	24.31	20.63	30.43	20.78
21.94	18.56	22.39	24.18	24.67	23.50	22.04	29.70	22.21	11.02	24.31	21.11	30.06	20.03
21.81	18.53	22.43	24.12	24.68	23.22	22.01	29.57	22.32	10.88	24.30	21.11	30.36	20.51
20.74	18.06	21.42	24.03	24.40	23.53	21.47	28.84	21.45	11.92	23.67	20.29	30.74	18.83
20.60	18.16	21.37	24.05	24.38	23.41	21.31	28.98	21.48	11.93	23.73	20.24	31.12	19.19
21.09	18.71	21.58	25.09	26.27	23.77	22.20	29.35	22.02	12.15	24.52	20.55	29.83	20.30
21.29	18.75	21.53	25.15	26.16	23.52	22.04	29.31	21.80	11.62	24.39	20.64	30.69	20.36
